# The Relationship between Animal Welfare and Farm Profitability in Cage and Free-Range Housing Systems for Laying Hens in China

**DOI:** 10.3390/ani12162090

**Published:** 2022-08-16

**Authors:** Shuai He, Jiao Lin, Qiongyu Jin, Xiaohan Ma, Zhongying Liu, Hui Chen, Ji Ma, Huancheng Zhang, Kris Descovich, Clive J. C. Phillips, Kate Hartcher, Zhonghong Wu

**Affiliations:** 1 State Key Laboratory of Animal Nutrition, College of Animal Science and Technology, China Agricultural University, Beijing 100193, China; 2 College of Animal Science and Technology, Hebei Agricultural University, Baoding 071001, China; 3 Pingliang Hongniu Institute, Pingliang 744000, China; 4 School of Veterinary Science, University of Queensland, Gatton, QLD 4343, Australia; 5 Institute of Veterinary Medicine and Animal Sciences, Estonian University of Life Sciences, Kreutzwaldi 1, 51014 Tartu, Estonia; 6 Curtin University Sustainability Policy (CUSP) Institute, Curtin University, Perth, WA 6845, Australia

**Keywords:** laying hens, animal welfare quality, cage rearing system, free-range rearing system, farm economics

## Abstract

**Simple Summary:**

With public concern increasing in China for animal welfare in livestock production, the welfare and economic outcomes of different farming systems for laying hens needs to be examined. Animal welfare outcomes and initiatives are linked to the economic efficiency of the farm, which for the producer may require a balance or trade-off between these issues. This study investigated animal welfare, egg production and economic parameters in cage and free-range farms in China. Our results identified potential welfare issues that can be addressed on both farm types, and potential points of income that can be improved by enhancing hen welfare. Therefore, our results offer some directions for welfare and economic improvements on cage and free-range egg farms in China.

**Abstract:**

Several countries and regions have regulations in place to provide standards for the welfare of production animals, which have implications for breeding, management and trade. In the chicken egg production industry, the welfare impacts of this are not well understood. In the past decades, free-range systems were widely used for local chicken breeds in poultry industry in China, but their use has gradually declined due to the lower competitiveness compared to commercial cage systems. However, the practices of free-range systems for hens raising have gradually increased again over the past decade, as consumer individualized demand for higher food quality and animal welfare has increased. We recruited 14 free-range farms and 45 cage farms from Beijing, Shandong, Hebei, Anhui, Yunnan, Gansu and Jiangsu provinces in China, for an evaluation of hen welfare, production and economic outcomes from farm operations. This study provides data for the welfare outcomes of laying hens in China and preliminarily explored the relationship between welfare level and economic income within farming system types. The researchers visited the farms and used Welfare Quality measures to investigate the welfare, and farm self-reported profits. Nonparametric Mann–Whitney U tests were used to compare the welfare scores between cage and free-range rearing farms. Correlation and regression are used for the analysis of the animal welfare scores, economic data, and production metrics. The general income from free-range farms was linearly correlated with red mite score and stocking density (*p* < 0.001 and *p* < 0.05, respectively). The results showed less centimeters of feeder and drinker space per animal in the free-range system than in cage systems (*p* < 0.05 and *p* < 0.01, respectively). Welfare scores for both the stocking density and beak condition were significantly better in the free-range systems than the cage systems (*p* < 0.001), as were qualitative behavior assessment scores (*p* < 0.05). The total egg production and peak egg production in cage farms were much higher than in free-range farms (*p* < 0.001), and egg loss rate was significantly lower (*p* < 0.001). While the production efficiency of free-range farms was lower than that of cage farms, general income per 10,000 hens was actually higher. Our results provide some evidence that some welfare indicators and general income (per 10,000 hens) in free-range farms in China were better than those of cage farms. The results indicate that better parasite control and lower stocking densities may result in improved hen welfare on free-range farms and potentially improve profitability. The level of welfare and economic benefits of free-range farms vary widely, and there was potential room for improvement in feeding space, drinking water space and human–animal relationship.

## 1. Introduction

The application of animal welfare principles has made a lasting impact on the livestock production industry since the initial development of the ‘Five Freedoms’ in 1965 [1,2]. Until now, the Five Freedoms, namely ‘freedom from hunger and thirst’, ‘freedom from discomfort’, ‘freedom from pain’, ‘freedom from injury and disease’, ‘freedom from fear and distress’, and ‘freedom to express most normal behavior’, have remained internationally understood as the general standards of animal welfare [3]. Relatively recently, the developments in the concept of animal welfare have extended past the avoidance of negative welfare states to focus more heavily on positive emotions and experiences of the animals, such as satiety, vitality, reward, contentment, curiosity and playfulness, in order to provide a “life worth living” [4]. Affective states, such as feelings and emotions, are not directly measurable, and therefore are commonly evaluated through the observations of animal behavior in specific contexts [5,6], or by using psychological assessments [7].

The scientific assessment of animal welfare is multi-dimensional, generally incorporating measures arising from animal physiology, anatomy, behavior, body condition and production [8,9]. The frameworks for welfare evaluation [10] are commonly comprised of parameters or domains of welfare evaluation with corresponding evaluation descriptions that provide sufficient information for assessing and improving the animal welfare outcomes. A comparison of animal welfare between different types of housing systems for animals does not only rely on the freedom to perform the most normal behaviors. The animals’ health and demeanor, as assessed by qualitative behavior analysis, are additional important considerations. Animal-based indicators may be preferred over resource-based indicators, if they can be measured over the large populations involved [11].

Optimal animal welfare relies on adaptative management and a good environment. A good environment requires investment in appropriate infrastructure, such as furnishings and perches [12], while adaptive management requires responsiveness to the needs of the flock over the lifetime of the hens [13]. In the laying hen industry, the systematic environment-based welfare assessment requires evaluation of multiple parameters, such as: enrichment provision; noise levels; stocking density; flock size; lighting; and air quality [14,15,16]. Similarly, reducing pathogenic microorganisms in the environment, providing sufficient space for animals to avoid stress, and ensuring adequate nutrition for sufficient immune responses are also critical aspects for maintaining welfare [17,18]. Kauselmann has reported that the addition of tasty straw pellets to pig diets was effective in reducing tail biting behavior [19]. Avoiding mortality due to disease outbreaks is not only fundamentally important for livestock welfare, but is also in the economic interests of the industry [20].

Egg production systems primarily use cage or cage-free systems. Over recent decades, the industry has largely moved towards more intensive systems [21]. The cages have production advantages in terms of feed conversion [22]. Yet in recent years, the use of cages in egg production has decreased and free-range breeding has increased, driven by the societal sensitivity for animal welfare [23,24]. In 2012, the European Union banned non-enriched cage systems for egg production, due to welfare concerns [25]. The small space allowance and high stocking density within the cage systems limits the ability of the hen to perform important natural behaviors and can increase stereotypical and other abnormal behaviors, including cannibalism [13]. Additionally, concentrations of harmful gases, such as ammonia and hydrogen sulfide, can be high, affecting hens’ endocrine system and leading to disease [26].

In 1985, China’s egg production surpassed that of the United States, ranking it first in the world [27]. Unlike Europe, rather than implementing a ban of the caged systems, China has encouraged the adoption of cage-free egg production. For example, in 2021, a large industry group, China Chain Store and Franchise Association, officially released a standard for its 1200 members and more than 460,000 stores [28], directing the production, circulation and consumption of cage-free eggs in China. As the scale of egg production in China remains the largest in the world [27], the systematic assessment of hen welfare in these systems is a priority issue.

In general, the industry development is limited or enhanced by resistance or support from consumers and the industry itself, with perceptions around the specific systems or the resulting products being a limiting factor for progress and implementation of higher welfare systems [29]. As an example, there is conflicting evidence that the different production systems result in differences in egg quality. The eggshell color affects consumer preferences [30]. The eggshell color is mainly controlled by the hen’s genotype [31], but the eggs from the cage production system have been reported to be darker in color, containing more protoporphyrin, mainly within the calcareous part of the shell [32]. The reported advantages in egg quality from the cage-free systems include egg weight, albumen levels and shell thickness [33]. The albumen may be greater in the eggs from birds in cages than those in floor pens [34]. In contrast, it has also been reported that several egg traits recorded from 20 to 60 weeks of age (yolk weight, yolk color and albumen ratio) were not greatly affected by the different housing systems (free-range versus cage) [35]. Therefore, the importance of the housing system for egg quality or color is evident for some of the characteristics, but not others.

The consumer interest in egg production systems is not limited to the welfare of the hens. An additional key concern for the public is with product safety [36]. The free-range hens foraging in outdoor enclosures have a higher risk of exposure to biosafety risks, compared to caged hens [37]. This can increase the occurrence of salmonella-contamination in the eggs and result in food safety problems [37]. In addition, the increase in stocking density and farm size has led to the occurrence and spread of salmonella in the laying flocks [38]. Different species of microscopic fungi have been detected on the eggshell surface of eggs produced in the different systems, and some microbiota on the surface may penetrate through the eggshell into the egg albumen [39].

The global consumers often have a keen interest in the practices surrounding the raising of livestock for animal products [40]. Most have a preference for livestock products from farms with high standards of animal welfare [41], although opinions on what constitutes good welfare may differ between the regions. For example, the participants from Canada overwhelmingly indicated a belief that farm animals’ welfare can be improved by using feeding systems that allow animals to display natural foraging and encourage contact with the natural environment [42]. They also reported being willing to pay a premium for animal products from the systems that support these conditions [42]. Most participants from Australia identified with respect for livestock emotions and low-stress handling of the animals [43].

The provision of resources and environments within the production systems has an economic impact on primary producers and may not always improve welfare. For example, hens in cage-free systems are typically provided with perches, foraging substrates and nest boxes. These can have welfare trade-offs as they allow for natural perching behavior but may increase the likelihood of keel fractures [44], and may also increase the risk of inhalable dust [12]. With a more variable environment stimulating a wide range of behaviors, the free-range systems increased the amount of activity and reduced feed conversion efficiency. Free-range farms often use low stocking densities, which demands more land investment [45], which leads to increased fixed costs per layer. Depending on the levels of the initial investment, the eggs from free-range systems are currently often more costly to produce than the eggs from cage systems and must command significant market premiums to be competitive [46]. Although many consumers express a willingness to pay more for eggs from a higher welfare system, the translation of this willingness into real-life purchasing decisions remains uncertain [42]. The current trend for a transition from cage to free-range systems needs further exploration of the impact on welfare and farm income. To better align the animal welfare innovations with the production input costs, insight is needed into the determinants that influence farm income in free-range and cage farms. We have conducted research on the level of welfare of laying hens in China in two housing systems, cage and free-range. The aim was to adapt a welfare grading system for domestic cage and free-range systems. The objective of this study was to determine the welfare outcomes and cost-benefits of these different laying rearing systems in China, as well as investigating the housing features within the housing types and how these relate to economic outcomes. The overall aim of this study was to understand the connection between animal welfare and production costs in the laying hen industry.

## 2. Materials and Methods

### 2.1. Participating Farms

**Figure 1:**
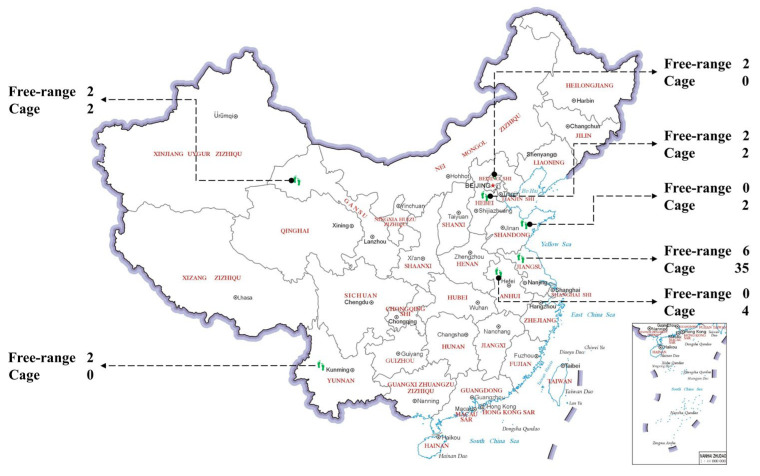
Distribution of participating egg production farms.

The requests were sent nationwide for farms to participate in this research; fifty-nine farms agreed. Forty-five of the participating farms used a cage system, ranging in scale between 12,000 and 1,800,000 hens. Fourteen of the farms used a free-range system, with between 3000 and 108,000 hens. The farms were located in the Chinese provinces of Beijing, Shandong, Hebei, Anhui, Yunnan, Gansu and Jiangsu (Figure 1). These farms were sent the economic and production parts of the survey in advance, and this was then followed by an on-site visit by a researcher for the welfare evaluation.

### 2.2. Description of the SURVEY

The data collection was conducted between May 2020 and June 2021. The questionnaire that was distributed to each farm for completion, prior to the on-site farm visits, comprised four parts: descriptive information about the farm; farm expenditure; farm production; and farm revenue.

The descriptive information included the name and address, date the farm was established, land area, farm composition (layer house, or both rearing and layer houses), source of hens (purchased, self-bred), layer breeds used, housing details for both rearing and laying (the number of nest boxes, nest size, floor space, number of henhouses, total stock, cage size), feeding method, water distribution method, manure removal method, waste disposal method, managers and technical staff information (number of permanent and temporary workers and support staff) and the sales channels for the eggs (specialized agricultural cooperative organizations selling on a commission basis, agricultural market, dealer with door-to-door sales, business order, supermarket, internet sales, other channel).

The section on the farm expenditure included questions about the farm’s investment in fixed assets (henhouses, nest boxes, perches, non-henhouse buildings, waterlines, drinkers, manure removal devices, wet curtains, fans, air conditioning, hot air stoves, underfloor heating, solar energy, heating, feed processing equipment, and egg collection equipment). The details of the variable costs were also obtained (pullets, feed, feed additives, litter, veterinary medicine, vaccines, third party biosecurity services, water, electricity and gas, coal, repair and maintenance, and tools and materials). The indirect costs (taxes, purchase of insurance, loan interest, handling fees, sales fees, transportation, marketing), labor costs, land rent, greening costs and other miscellaneous expenses were also obtained.

The section on farm production included questions on key performance indicators (volume of egg production, production cycle, raising period, feed intake, average egg weight, length of peak production, egg loss rate, feed conversion ratio).

The farm revenue was determined from the price at the farmgate and annual sales for the main product—eggs—and any secondary products, such as spent hens and manure for fertilizer. The gross profit was calculated as the farm revenue minus the total expenditure. All of the data from the key economic indicators were collected and converted into units of 10,000 RMB per 10,000 birds.

### 2.3. Animal Welfare Assessment

Three animal production scientists from the China Agricultural University, two animal welfare scientists from the University of Queensland and one animal welfare scientists from Curtin University in Australia formed an expert consultation panel in order to establish the weighting of the evaluation index of layer welfare, making reference to prior literature [47,48,49]. Included in this process, two domestic (Chinese) standards and one international (European) standard were referenced for the setting of each welfare score and the weighting of the welfare scores. These standards were the China Association for Standardization of Farm Animal Welfare Requirements: Laying Hen T/CAS 269-2017 [50], a local standard of Tianjin ‘Construction and Feeding Standard of Scattered Chicken Farms’ and DB12/T 754-2017 [51], and the EU regulation 1999/74/EC [52].

The methods for evaluating the welfare of hens in our survey was adapted from the Welfare Quality Assessment Protocol for Poultry (Table 1) [53,54]. In situ observations were completed on each farm by two researchers (Jiao Lin and Qiongyu Jin) who were trained by the expert consultation panel. Hard copy recording sheets were used to record 18 items relevant to hen welfare at each of the farms. These were feeder space, drinker space, perch availability, evidence of red mites, a ‘dust sheet’ test, the presence of thermoregulatory behavior such as panting and huddling, stocking density, toe damage, mortality rate, observable clinical conditions such as eye disease, respiratory tract infection, micro enteritis and comb-like abnormalities, beak trimming practices, feather damage, use of nest boxes, use of litter, enrichment measures, an ‘avoidance distance’ test (ADT), and a qualitative behavior assessment (QBA). The ADT is a test that measures fear behavior in response to humans [55], generally using an unfamiliar person. We observed the behaviors of hens from all of the farms and assessed them against 20 qualitative descriptors according to the Welfare Quality Assessment Protocol for Poultry [53], before combining them into an overall QBA score. These 20 qualitative behaviors were scored by emotional determination, including active, relaxed, comfortable, fearful, agitated, confident, depressed, calm, content, tense, unsure, energetic, frustrated, bored, friendly, positively occupied, scared, nervous, happy and distressed. The 18 welfare indicators were partitioned into one of four parameters: (1) raising; (2) henhouse; (3) health condition and (4) appropriate behavior [56].

### 2.4. Data Analysis

The score for each animal welfare indicator was multiplied by the allocated weighting factor to calculate a total welfare score for each farm (Table 1). Nonparametric Mann–Whitney U tests were performed in SPSS software (version 25; IBM, Chicago, IL, USA) to compare the welfare scores between the cage and free-range farms. Spearman correlation coefficients between the economic data matrix and welfare scores were calculated, using GraphPad Prism v7.0. Linear regression model and inverted U-shaped models relating welfare scores to the gross profit and general income were constructed, using the ‘basicTrendline’ package in R software.

## 3. Results

**Figure 2:**
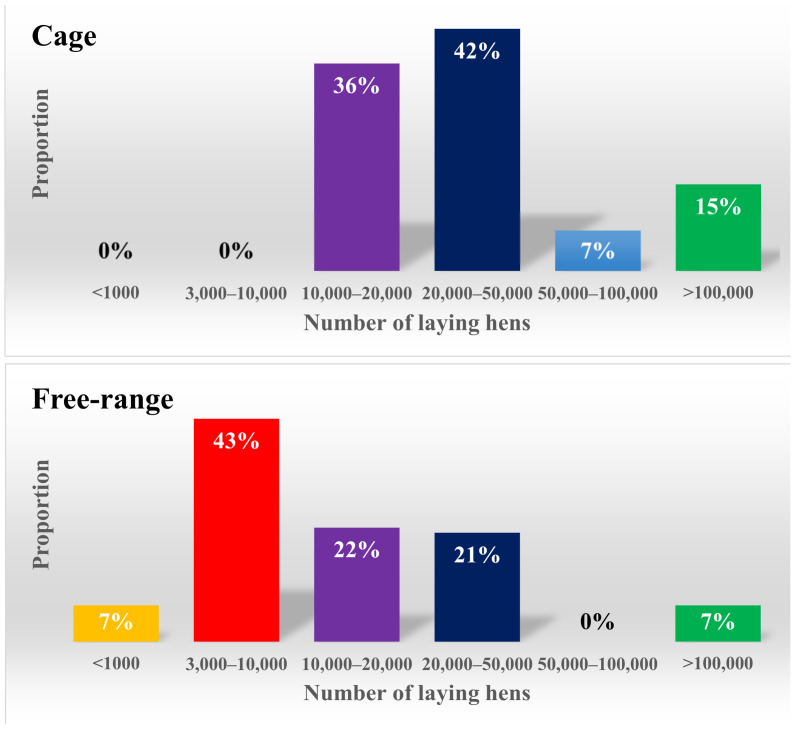
Number of hens in participating cage and free-range farms.

The number of hens in the participating free-range farms in our study mainly ranged from 3000 to 50,000, while the number of hens in cage farms with caged systems mostly were mainly in the range from 10,000 to 50,000 (Figure 2).

The outdoor areas of the free-range farms were covered with vegetation and the landscapes were both mountainous and flat, depending on the farm. The cage farms all used one of two types of cage systems: ladder or stacked cages, and most used automated feeding, egg collection and manure removal systems.

### 3.1. Development of the Scoring Standard for Welfare

In the investigation process, the main welfare reference was the Welfare Quality [53] method. We redefined the applicable weightings and scoring criteria based on our research of domestic egg farms for both cage and free-range hens. The indicator scores ranged from 0 to 10, divided into a six-point scale, with 0 representing the worst welfare and 10 the best welfare (Table 2 and Table 3). The welfare indicators (*n =* 18) were divided into four evaluation parameters: raising; henhouse; health condition and appropriate behavior, and both the parameters and the indicators within each parameter were assigned a weighting of their contribution to an overall farm welfare score (Table 1).

### 3.2. Welfare Evaluation Results

**Figure 3:**
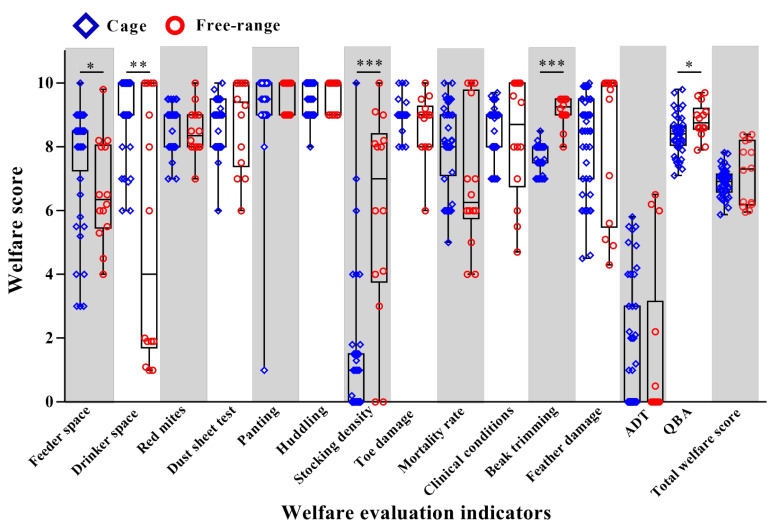
Welfare indicators scores for laying hen farms using caged and free-range systems. Note: ADT = avoidance distance test; QBA = qualitative behavior assessment; Significant differences are denoted by * *p* < 0.05; ** *p* < 0.01, and *** *p* < 0.001 using Nonparametric Mann–Whitney U tests.

The feeder space in the surveyed farms was mostly distributed between 5 cm and 10 cm per bird for the free-range farms, and between 10 cm and 15 cm per bird for the cage farms (Figure 3). All 45 cage farms and 43% (6/14) of the free-range farms used nipple drinkers. No more than 10 hens in any cage farms were assigned to a single nipple. Among the six free-range farms using this drinker type, two supplied only one nipple for more than 15 hens, while three farms had no more than two hens using one nipple. Eight of the free-range farms used grooved water equipment and of these, five farms gave less than 1 cm of drinker space per hen on average, while the other three farms gave more than 5 cm of drinker space per hen, with the largest giving 18 cm per hen. In terms of stocking density, 38% percent (17/45) of the cage farms provided less than 450 cm^2^ space for each hen, and only 13% percent (6/45) of the caged hens were provided more than 550 cm^2^ space per hen. A total of 50% percent (7/14) of the free-range farms had an indoor density of less than nine hens/m^2^, 43% percent (6/14) of the free-range farms had an outside density of less than nine hens/m^2^.

There was no overall significant difference in the total welfare scores of birds in the cage and free-range farms (*p* > 0.05; Figure 3). However, differences were evident for the individual welfare indicators. The welfare score for beak trimming was significantly better for the free-range farms compared to the cage farms (*p* < 0.001). There was evidence of light to moderate beak trimming on all 14 of the free-range farms and no abnormalities. Beak trimming was undertaken at all of the cage farms at 1 day of age. There was a higher proportion of slight abnormalities in the cage hens than in free-range hens, but no influence on feeding was found (Figure 3).

The feeding and drinker space scores were greater for the birds in the caged than the free range farms (*p* < 0.05 and 0.01, respectively; Figure 3), but the welfare scores for stocking density were significantly lower (*p* < 0.001). More than 85% of the cage farms (39/45) scored < 2 points for stocking density, even though the stocking density scale was scaled according to the farm type. The qualitative behavior assessment (QBA) scores were significantly better for the birds on the free-range farms than for those on the farms with cages (*p* < 0.05; Figure 3). There were no significant differences between the scores for red mites, the dust sheet test, panting and huddling behavior, toe damage, mortality rate, clinical conditions, feather damage and the ADT between free-range and cage farms (all *p* > 0.05; Figure 3). Most of the farms scored well on all of these indicators, except for mortality rate and feather damage, which showed considerable farm variation, and the ADT test, for which the scores were low for the birds in both of the farm systems (Figure 3).

**Figure 4:**
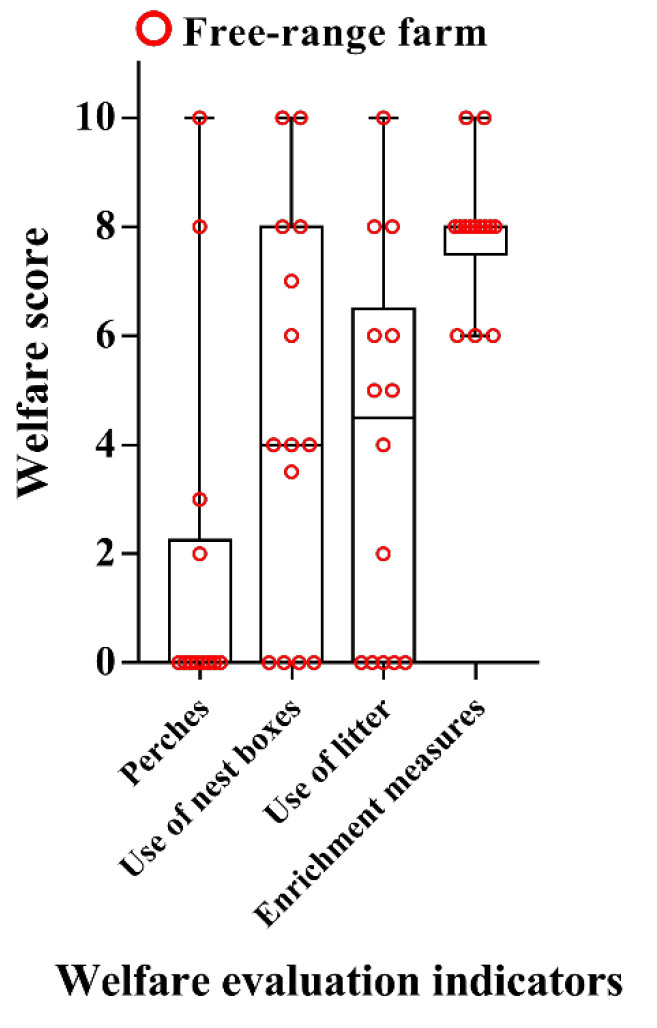
Scores for welfare indicators found only on free-range egg production farms.

The scores for perch provision and utilization on the free-range farms were particularly low, with more than 70% (10/14) of farms receiving a score of 0 (Figure 4). The scores for nest boxes and litter varied widely between the free-range farms (Figure 4). The scores for enrichment measures were generally high, ranging between 6 and 10 for all of the farms. None of the participating cage farms provided perches, nest boxes, litter or enrichment, therefore receiving 0 points for these items.

### 3.3. Economic Performance

**Figure 5:**
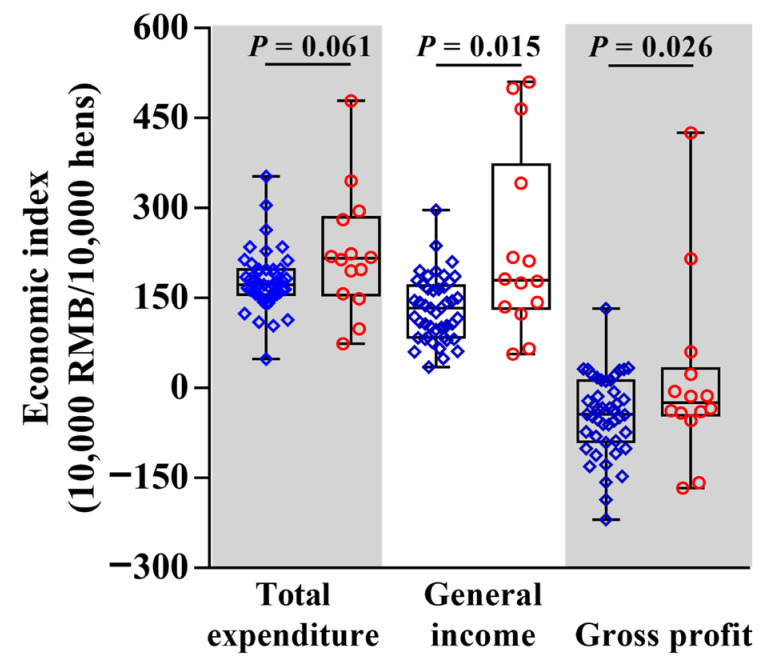
Total expenditure, general income and gross profit of cage and free-range laying hen farms comparison using a Nonparametric Mann–Whitney U tests.

**Figure 6:**
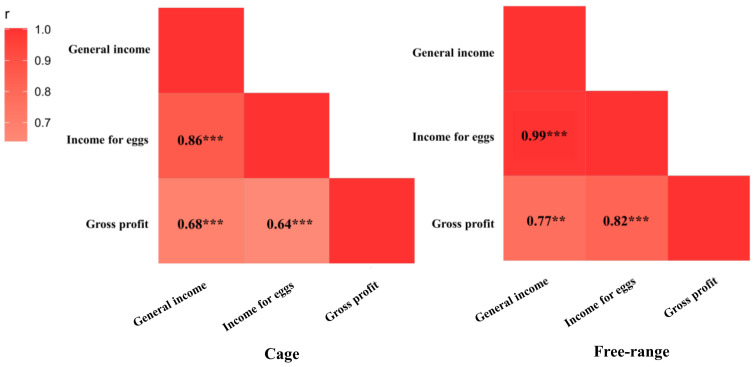
Spearman correlations between general income, income for eggs and gross profit. Note: Significant differences are denoted by ** *p* < 0.01, and *** *p* < 0.001.

**Figure 7:**
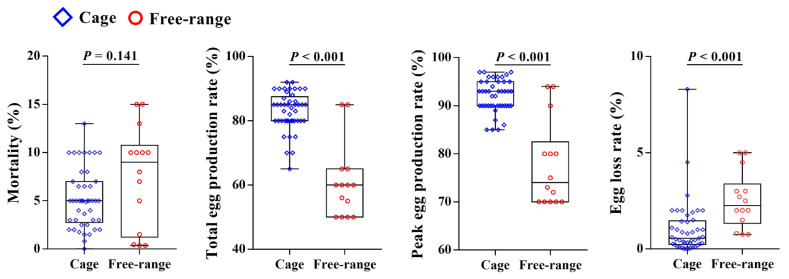
Key production data comparison using a Nonparametric Mann–Whitney U tests in laying hen farms with different feeding modes.

There was no difference in expenditure between the cage and free-range farms (*p* = 0.06; Figure 5). The income and profit were both significantly greater for the free-range farms than the cage farms (*p* =0.015 and *p* = 0.026, respectively; Figure 5). The income from the egg sales was strongly correlated with general income and gross profit for both the cage farms and free-range farms (Figure 6). The analysis of the egg production indicators directly related to the economic data found that the total egg production and length of peak egg production in cage farms was greater than for the free-range farms (*p* < 0.001; Figure 7). The egg loss rate for the cage farms was significantly lower than that for the free-range farms (*p* < 0.001). There was no difference between the cage and free-range farms in mortality rate (*p* = 0.14; Figure 7).

### 3.4. Relationships between Welfare and Economic Indicators

**Figure 8:**
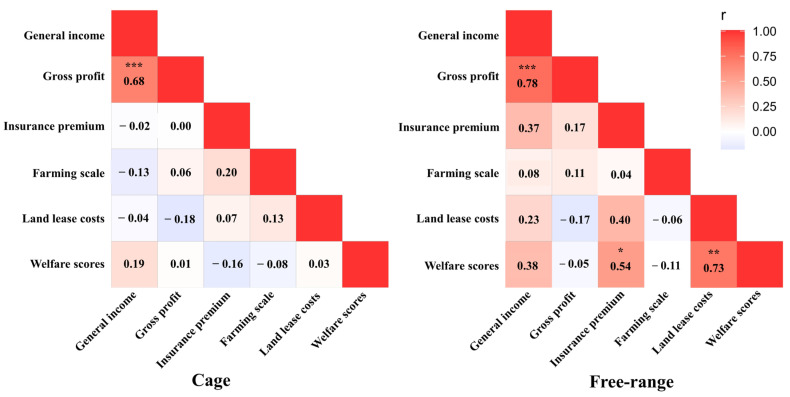
Spearman’s correlations between economic data and overall welfare scores of cage and free-range egg production farms. Note: Significant differences are denoted by * *p* < 0.05; ** *p* < 0.01, and *** *p* < 0.001.

In the cage farms, no significant correlations were found between the welfare scores and a range of economic indicators (*p* > 0.05; Figure 8). The welfare scores from the free-range farms had positive correlations with the insurance premium costs (*p* < 0.05) and land lease costs (*p* < 0.01; Figure 8). The general income had a positive correlation with gross profit for cage and free-range farms (*p* < 0.001; Figure 8).

**Figure 9:**
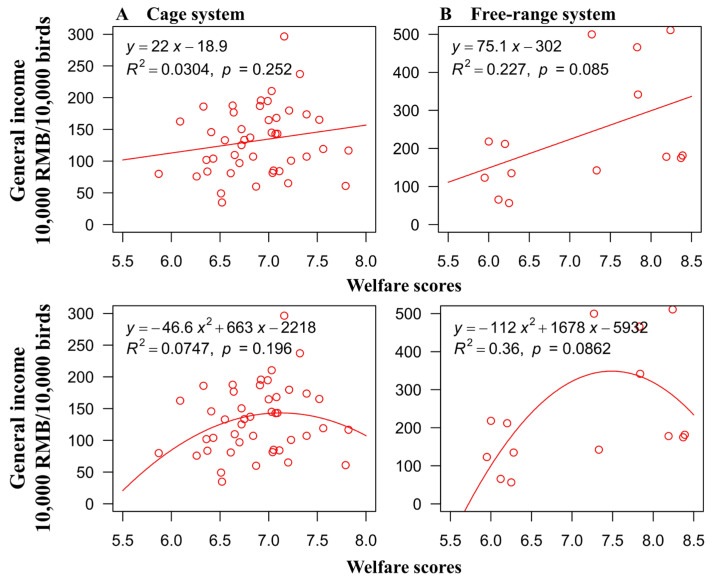
Regression model for a relationship between general income of cage (**A**) and free-range (**B**) egg production and overall animal welfare score.

The data from all 14 free-range farms and 45 cage farms were used to develop the regression equation for each system. For the free-range farms, the results of the linear regression model showed no relationship between the welfare scores and general income (*p* = 0.085; Figure 9). Non-linear regressions were not significant for the general income on the free range farms to be curvilinearly related to welfare (*p* = 0.086; Figure 9).

**Figure 10:**
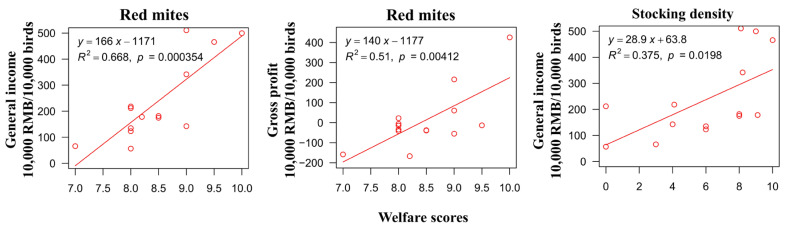
Linear regression relationship between economic indices for free-range farms and score for welfare indicators.

There was a significant positive relationship between the general income and the red mite score (*p* < 0.001) and the general income and the stocking density (*p* < 0.05), and a significant positive relationship between the gross profit and the red mite score in the free-range farms (*p* < 0.01; Figure 10). There was no linear relationship between the other welfare indicators and the economic index, regardless of the farm type (*p* > 0.05).

## 4. Discussion

The goal of this study was to compare cage and free-range egg farms in China in terms of animal welfare and to adapt a welfare grading system for domestic cage and free-range systems. This study also aimed to provide a preliminary analysis of the economic implications of different systems in order to identify the potential welfare items that can be value-added and the aspects of income that can be improved through welfare improvements.

The participating cage farms provided more feeder space per hen than the free-range farms. The feeder space on the cage and free-range farms met the minimum requirements from the European Union 1999/74/EC [52]. In the conventional cages, the hens given less feeder space were reported to spend less time feeding, which can lead to wasted feed and poorer feed conversion [57]. Synchronized feeding, in line with diurnal rhythms, is considered important for the welfare of hens [58]. The different production systems, facilities and environmental influences differ in the synchronization rate of feeding in hens [59]. Therefore, the results of this study may suggest that feeding competition may be higher in the free-range systems, due to reduced feeder space per hen. It would be useful to quantify through future research the number of the free-range hens that were seen eating simultaneously, and the behavioral evidence of restricted feed access or resource competition. Most of the surveyed farms used nipple systems for providing water, which save water and reduce microorganism contamination [60]. The drinker space per hen on caged farms ranged from 10 hens/drinker at the lower end to the three hens/drinker at the highest. In contrast, the free-range farms were polarized in their drinker provisions per hen, with some being very high and others very low, suggesting that more professional guidance is needed around an adequate water supply for these farms.

The QBA index has been widely used to assess the welfare and emotional states in a variety of species: sheep, goats, pigs, giraffes and hens [61,62,63,64]. The QBA scores for the hens in the free-range systems were significantly better than for the hens in the cage systems. This suggests that the birds in the free-range systems are considered to have more positive affective experiences than those in cage systems. On some behavioral measures (huddling and panting) the results were very similar between the two farm types. These behaviors are indicative of inappropriate ambient temperatures, with huddling occurring in cold conditions [65] and panting in hot [66], suggesting that the temperatures on both of the farm types met the needs of the birds, at least at the time of the data collection. In contrast, the performance of the hens in the ADT was poor in both cage and free-range systems. The ADT testing is usually completed by strangers, for the hens. However, the recent research suggests that hen behavior in response to a stranger may not be reflective of the stockperson–animal relationship [67]. The birds on the surveyed farms from both of the systems had little experience with strangers, which is likely to have influenced their ADT results. However, the animal–stockperson relationship is recognized to be highly influential for farm animal welfare [68]. Therefore, future research that measures ADT with both a stranger and a familiar stockperson may provide useful insights into hen welfare and the ADT methodology.

For the analysis of economic performance, there were no significant correlations found between the overall welfare score and any economic indicator for the cage farms. However, the overall welfare score for the free-range systems was positively correlated with insurance costs and land lease costs. The livestock insurance, which pays for the death and abortion of animals not caused by human factors, contributes to the healthy development of the livestock sector by supporting good animal welfare, particularly in regards to animal health, as found for dairy cows in Turkey [69]. The insurance companies usually appoint professional veterinarians to review the claims data provided by the farm before providing compensation, as found for broiler farms in Iran [70]. An important basis for the insurance companies to assess claims is that the death of the animal was not caused by the farmer’s negligence and mismanagement [71,72]. In China, some of the government web portals publish information on egg farming insurance providers, types of insurance and principles of compensation treatment, with the aim of increasing farmers’ resilience to the egg farming risks [73]. In our survey, the farmers also mentioned that the insurance companies review the farm insurance conditions, including the health, welfare and environmental conditions of the hens, before insurance agreements are reached on the farm. The projects evaluated by insurance companies often obtain the attention of the farmers, which include hen welfare, and this could be a potential reason why the welfare score of the surveyed free-range farms are positively correlated with the cost of insurance.

The welfare scores were fitted against the key indicators of economic performance for all of the surveyed farms, using a variety of potential association curves. Only weak inverted U-shaped curves were able to be fitted, and these had relatively low correlation coefficients. This finding differs somewhat from a study of Danish pig farms that found (under the same conditions) relationships between the level of animal welfare and economic performance showed an inverted U-shaped relationship [74]. The rising phase of the inverted U-shaped curve occurs as improvements in animal welfare reduce the mortality, which in turn increases the economic performance [74]. The mortality rate of the free-range farms showed no difference compared with that of cage farms (Figure 7), which may lead to the different distribution of the two system types in the inverted U-shaped model. Even with one housing system for hen-raising systems, the differences in the management levels between the farms can affect the relationship between animal welfare and economic performance [74].

A linear relationship between the welfare indicators and general income points to the fact that the implementation of some of the welfare improvements will contribute to farm profitability. For the free-range farms in this study, a lower number of red mites and lower stocking densities were positively correlated with the general income in the surveyed farms. While a causal relationship cannot be determined from this analysis, the results suggest that better parasite control and lower stocking densities may help free-range farms in China improve profitability. The red mites damage to the housing environment caused by toe pecking between hens can lead to considerable animal losses [75]. The long-term infestation by red mites impacts on hen health, reduces their production performance and negatively impacts egg quality [76,77,78]. The laying hens may act as vectors of infection for the red mite pathogens [79], which may explain the positive correlation between the red mite scores and economic returns on the free-range farms in the current study. The relatively low stocking densities may reduce competition for space and feeding, improve the survival time of hens and improve the lifetime egg production [80].

The income from the egg sales was significantly correlated with general income in both the cage and free-range farms. The total egg production and peak egg production rate of the cage laying hens were significantly higher than those of the free-range laying hens, and the egg loss rate was significantly lower. Our survey on the egg loss rates between the different rearing systems appears to be inconsistent with the findings from previous studies, which found that the free-range systems have lower breakages compared to the cage system [81]. However, egg loss rate can occur due to several different factors, including nest box use. In the surveyed free-range farms, some of the eggs were observed outside the nest box. When the eggs are found outside the nest box, but the time of production cannot be determined, the eggs are abandoned, resulting in egg loss, which may explain the results in the current study. The egg production on the cage farms was higher than that of the free-range farms, which is consistent with previously published research [82]. This may be due to reduced feed intake caused by the environmental instability of the free-range laying hens [22].

It is generally recognized that welfare is more variable on the free-range farms than cage farms where it is generally low, because of different resourcing standards [45]. Similarly, the cage farms are relatively close in terms of welfare input levels, so they are more concentrated in the welfare economy curve. Similar to the results of Sherwin’s assessment of hen welfare in different housing systems, the cage and free-range systems provided both positive and negative welfare aspects for the hens [83]. From our evaluation results, there was no significant difference in the overall welfare levels between the cage and free-range systems. Therefore, a comparison of the physical health and physiology of the hens from different housing systems after slaughter would refine the results of the animal welfare evaluation. Unfortunately, due to farm biosecurity control in other areas of the country, we did not have the opportunity to visit more of the farms in this project, which would have allowed more complex welfare–economic interactions to be tested.

Chicken keeping and rearing in China has occurred for thousands of years [84]. Domestic animals were traditionally raised in backyards [85], where the traditional egg farming was very small scale. Nowadays, China has a large population and an extremely large egg production industry [27]. The scale of egg farming is also gradually expanding. Globally, the public in general is becoming more conscious of the issues around animal welfare and support for the modern free-range model is emerging [86]. Our results suggest that the level of welfare and economic outcomes of both of the egg-production farm types in China vary widely. The relationship between the economic data and welfare levels in the free-range system should be further explored.

## 5. Conclusions

Among the farms investigated in the current study, the free-range farms had lower feeder space and drinker space for the hens than the cage farms, but had welfare advantages in lower stocking densities, better beak condition, fewer red mites and better Qualitative Behavior Assessment scores, indicating an improved affective state. The egg production rate of the free-range farms was lower than that of the cage farms, however general income per 10,000 hens was higher. The total welfare scores were comparable between farm type. However, differences were found between the individual welfare indicators. The results indicate that better parasite control and lower stocking densities may result in improved hen welfare on the free-range farms and potentially improve profitability. The hen welfare in the cages could be improved through lower stocking densities and environmental enrichment. The level of welfare and the economic benefits of the free-range farms vary widely, and many farms of both production types have someroom for improvement in welfare.

## Figures and Tables

**Table 1:** The evaluation method and weightings for welfare indicators in the calculation of farm welfare scores.

Welfare Parameter	Weight	Welfare Evaluation Indicators	Indicator Weight	Evaluation Method [53]
Raising	20%	Feeder space	10.0%	Calculated by the total length of available feeders.
		Drinker space	10.0%	Calculated the total space of available drinkers in the house according to drinker type.
Henhouse	21%	Perches	1.0%	Recorded if any of the perches have sharp edges.
		Red mites	4.5%	Evidence of red mites under perches, or in cracks and crevices.
		Dust sheet test	5.0%	^a^ one dustproof paper placed near the house entrance and one placed in the middle of the shed for 5 min.
		Stocking density	10.5%	Ratio of total space in the house that is permanently accessible for the birds in relation to total number of hens.
Health condition	39%	Toe damage	8.0%	Both feet of 100 randomly selected hens were examined.
		Mortality rate	12.0%	Death records were collected.
		^b^ Clinical conditions	4.0%	100 hens were randomly selected and observable clinical conditions ^a^ noted.
		Beak trimming	6.0%	100 hens were randomly selected and beak condition noted.
		Panting	4.5%	Proportion of hens panting in the front, middle and back of the house.
		Huddling	4.5%	Percentage of huddling during a flock walk at the start, halfway point, and end of the assessments.
Appropriate behavior	20%	Feather damage	3.0%	100 hens were randomly selected and feather damage noted.
		Use of nest boxes	2.0%	With/without nest boxes and establish the distribution of eggs over rows and nest boxes.
		Use of litter	1.0%	Observe birds performing dust bathing behavior in loose friable material.
		Enrichment measures	2.0%	Checked the area inside and around the henhouse for enrichment.
		Avoidance distance test (ADT)	5.0%	Twenty-one chickens were randomly selected for the ADT evaluation.
		Qualitative behavior assessment (QBA)	7.0%	5-min in situ behavior observations in four locations of the house using qualitative descriptors.

Note: ^a^ dustproof paper = a sheet of black A4 size paper; ^b^ clinical conditions including to the observable indicators of eye disease, respiratory tract infection, enteritis and comb-abnormalities.

**Table 2:** Scoring for environmental indicators.

Scores	10 Points	8 Points	6 Points	4 Points	2 Points	0 Point
Feeder space (cm/bird)	>15	10	5	4	3	<1
a Drinker space Nipple availability (animal/per nipple)	3	5	10	12	15	20
^b^ Drinker space Groove type (cm/bird)	10	5	2.5	2	1	<0.5
Perches	More than 50% of the perches are located in the rest area and their cross sections do not have sharp edges	More than 50% of the perches are located in the rest area but some cross sections have sharp edges	Rest area perches less than 50% and cross sections without sharp edges	The rest area perch is less than 50% but its cross section has sharp edges	Few perches in the rest area	No perches
Red mites	No red mites in the henhouse, no spider webs on the doors and windows or no ^c^ parasites	A small number of spider webs on doors or windows or parasites found in chicken coop	Red mites are found on chickens or in coop, but are not visible in large numbers	Many spider webs or evidence of parasites was found in the chicken coop	Red mites were found in large numbers in the henhouse	In the henhouse, spider webs were densely distributed, red mites were rampant (i.e., there were a large number of red mites)
Dust sheet test	Completely dust-free	A small amount of dust	More than half of the dustproof paper covered with dust	Covered with a layer of dust	Covered with a lot of dust	The color of the paper was obscured by dust
Cage density (cm^2^/bird)	>660	618	576	534	492	<450
Indoor and outdoor stocking density (animal/m^2^)	Inside: <9 Outside: <0.5	Inside: 9–11 Outside: <0.5	Inside: <9 Outside: 0.5–2	Inside: 9–11 Outside: 0.5–2	Inside: >11 Outside: 0.5–2	Inside: 9–11 Outside: >2

Note: ^a^ Drinker space Nipple availability represents the scoring criteria for nipple drinking equipment; ^b^ Drinker space Groove type represents the scoring criteria for groove type drinking equipment; c Parasites refer to beetles, lice, worms, flies, spiders; Scoring methodology has been referenced from the Welfare Quality Assessment Protocol for Poultry [53].

**Table 3:** Scoring for health condition and behavioral indicators of hen welfare.

Scores	10 Points	8 Points	6 Points	4 Points	2 Points	0 Point
Clinical conditions	0%	<1%	1%	5%	10%	>20%
Toe damage	0%	<1%	1%	5%	10%	>20%
Mortality rate	<1%	5%	10%	15%	20%	>25%
Panting	0%	1%	3%	5%	10%	>20%
Huddling	0%	1%	3%	5%	10%	>20%
Beak trimming	The beak is intact and there are no abnormalities	Light or moderate trimming but no abnormality	Mild or moderate trim but slightly abnormal	The beak is obviously abnormal, but this has little effect on feeding	Abnormal beak with effect on eating	Beak completely deformed
Feather damage	No or slight wear, plumage nearly complete	One of the head, neck, abdomen or dorsum is damaged and less than 5 cm in diameter	Multiple feather damage on head, neck, abdomen or back and back legs and less than 5 cm in diameter	There is a featherless area in one of the head, neck, abdomen, or dorsum where the diameter damage is greater than 5 cm	There are many featherless areas on the head, neck, abdomen or back and buttocks, i.e., the diameter damaged is greater than 5 cm	The feathers were badly damaged and there were multiple skin lesions
Use of nest boxes	There are nest boxes evenly distributed in the house, and eggs evenly distributed in the nest boxes	The nest boxes are evenly distributed in the house but the eggs are not evenly distributed in the nest boxes	There are nest boxes but they are not evenly distributed throughout the house	There are nest boxes but eggs can be seen outside the nest boxes	There are nest boxes but quite a few of the eggs are outside the nest boxes	There are no nest boxes
Use of litter	Two or more hens take a sand bath together	There are bedding layers for sand bathing	No hens sandbathe but most use bedding	A small percentage of hens use bedding	Bedding is available but rarely used by hens	No bedding present
^a^ Enrichment measures	More than 75% of hens using	50%–75% of hens using	25%–50% of hens using	Less than 25% of hens using	No hens using enrichment measures	No enrichment present
Free-range ^b^ ADT	25 cm	50 cm	75 cm	100 cm	125 cm	150 cm
Cage ADT	10 cm	20 cm	30 cm	40 cm	50 cm	60 cm

Note: ^a^ Enrichment measures include hanging ropes, bales of hay, partitions, roofs in free range area; ^b^ ADT = avoidance distance test; Scoring methodology has been referenced from the Welfare Quality Assessment Protocol for Poultry [53].

## Data Availability

The data presented in this study are available on request from the corresponding author. The data reflect the specific conditions of agricultural enterprises that are covered by the privacy policy.
